# Aquaporin-10 Represents an Alternative Pathway for Glycerol Efflux from Human Adipocytes

**DOI:** 10.1371/journal.pone.0054474

**Published:** 2013-01-29

**Authors:** Umberto Laforenza, Manuela F. Scaffino, Giulia Gastaldi

**Affiliations:** Department of Molecular Medicine, University of Pavia, Pavia, Italy; Wageningen University, The Netherlands

## Abstract

**Background:**

Glycerol outflow from adipocytes has been considered for a decade to be mediated by aquaporin-7, an aquaglyceroporin highly expressed in the adipose tissue. Its involvement in glycerol metabolism has been widely studied also in humans. Recent studies in different aquaporin-7 KO mice models pose two different questions 1) the exact localization of aquaporin-7 in human white adipose tissue; 2) the existence of other aquaglyceroporins that work with aquaporin-7 to guarantee glycerol efflux and thus a normal adiposity in humans. To this purpose we investigated the expression, the localization and the functioning of aquaglyceroporin-10 in subcutaneous white adipose tissue, in isolated and cultured differentiated adipocytes.

**Methodology/Principal Findings:**

Aquaporin-7 and -10 were expressed in the white adipose tissue both at mRNA and at protein level. Immunofluorescence revealed aquaporin-7 and -10 labelling in the human adipose tissue both to the plasma membrane and to a thin rim of cytoplasm of adipocytes. Aquaporin-7, but not aquaporin-10, colocalized with the endothelial marker CD34. Human cultured differentiated adipocytes showed an aquaporin-7 and -10 labelling mainly in the cytoplasm and in the lipid droplets with insulin reinforcing the lipid droplets staining and isoproterenol inducing its translocation to the plasma membrane compartment. Water and glycerol permeability measurements using adipocytes and adipose membrane vesicles confirmed the presence of functioning aquaglyceroporins. Aquaporin-10 silencing in human differentiated adipocytes resulted in a 50% decrease of glycerol and osmotic water permeability.

**Conclusions/Significance:**

The results indicate that aquaporin-7, differently from mice, is present in both adipocyte and capillary plasma membranes of human adipose tissue. Aquaporin-10, on the contrary, is expressed exclusively in the adipocytes. The expression of two aquaglyceroporins in human adipose tissue is particularly important for the maintenance of normal or low glycerol contents inside the adipocyte, thus protecting humans from obesity.

## Introduction

When fasting and exercising, adipose tissue triglycerides are hydrolyzed into glycerol and free fatty acids, and both products are released into the blood stream by different transport mechanisms [1], [2]. Glycerol efflux from adipocytes occurs via a specific glycerol channel that belongs to the aquaporin (AQP) family, called AQP7 [2], [3]. AQPs are integral membrane proteins that operate as water channels. So far 13 AQP homologues have been identified in mammals, and divided in three groups, based on their functional characteristics,: i, orthodox AQPs (AQP1, -2, -4 and -5) selectively permeable for water; ii, aquaglyceroporins (AQP3, -7, -9 and -10) that are also permeable for glycerol, urea and other small solutes as well as water; iii, unorthodox aquaporins (AQP6, -8, -11 and -12), whose peculiar intracellular localization and functions are currently being researched [4]–[6].

AQP7 is abundantly expressed in mammal adipose tissue where represents the traditional pathway for glycerol transmembrane flow. In rodents, AQP7 is up-regulated by fasting, low insulin, peroxisome proliferators-activated receptor alpha and gamma (PPARα and γ), down-regulated by feeding, high insulin, dexamethasone, while β-agonists effects are less clear [3], [7]–[11]. A notable impulse in the study of AQP7 role in adipose tissue was given by the results of two independent studies with AQP7 null mice [12], [13]. AQP7 deficient mice show marked adipocyte hypertrophy and develop adult-onset obesity and insulin resistance. It has been suggested that glycerol entrapped into AQP7 null adipocytes rises in concentration and increases triglycerides synthesis and accumulation by inducing the glycerol kinase activity. Similar results were obtained with AQP7-knockdown 3T3-L1 adipocytes [13]. All together, data obtained in murine KO models for AQP7 gene have suggested a pivotal role of AQP7 in maintaining the normal adiposity and that its altered expression might be implicated in the susceptibility to obesity and related disorders [14]. In contrast, two studies obtained with others AQP7-KO mice models did not show any difference in adipocyte cell volume and in adipose tissue mass [15], [16].

Further studies on AQP7 gene expression in human adipose tissue demonstrated a down-regulation in obese vs. lean subjects [17], [18]. Prudente et al. [19] identified a common polymorphism in the promoter region of the human AQP7 gene (A-953G SNP) that could be related to obesity and type 2 diabetes. Differently from mice, AQP7 involvement in human obesity is far from been clearly defined. Surprisingly, two studies analyzing the genetic mutations of AQP7 in human subjects did not find any correlation with obesity or type 2 diabetes even in subjects with homozygous G264V mutant, encoding for a not functional protein [17], [20]. The apparent differences between results in developing obesity obtained in KO mice and homozygous G264V mutant subjects suggest the presence of another glycerol pathway in human adipocytes.

Recently, AQP3 and 9 have been also identified in the human adipocytes and their function in transmembrane glycerol movement demonstrated [11].

Here we focused our attention on AQP10, an aquaglyceroporin expressed only in the human but not in the mouse small intestine where it has been demonstrated to be a pseudogene [21]–[24]. We have searched for AQP10 expression and the eventual localization of the protein in human subcutaneous adipose tissue, under the hypothesis that it could be an alternative pathway for glycerol efflux from human adipocytes.

In particular in this study we examined: 1) the expression of AQP7 and 10 mRNA in human subcutaneous adipose tissue, in isolated and differentiated human adipocytes by RT-PCR; 2) the AQP7 and 10 protein expression by immunoblotting of plasma membrane preparations; 3) the cellular localization of both AQPs by immunofluorescence; 4) the possible regulatory role of insulin and isoproterenol; 5) the water and glycerol permeability of isolated adipocytes and adipocyte plasma membrane vesicles measured by a stopped-flow light scattering method; 6) the effect of AQP10 silencing on the water and glycerol permeability of human differentiated adipocytes.

The results reported here provide evidence that AQP10 is another glycerol channel expressed in the plasma membrane of human adipocytes.

## Results

### Real Time RT-PCR (qRT-PCR) Analysis of AQP7 and 10 mRNA Expression in Human Adipose Tissue

**Figure 1:**
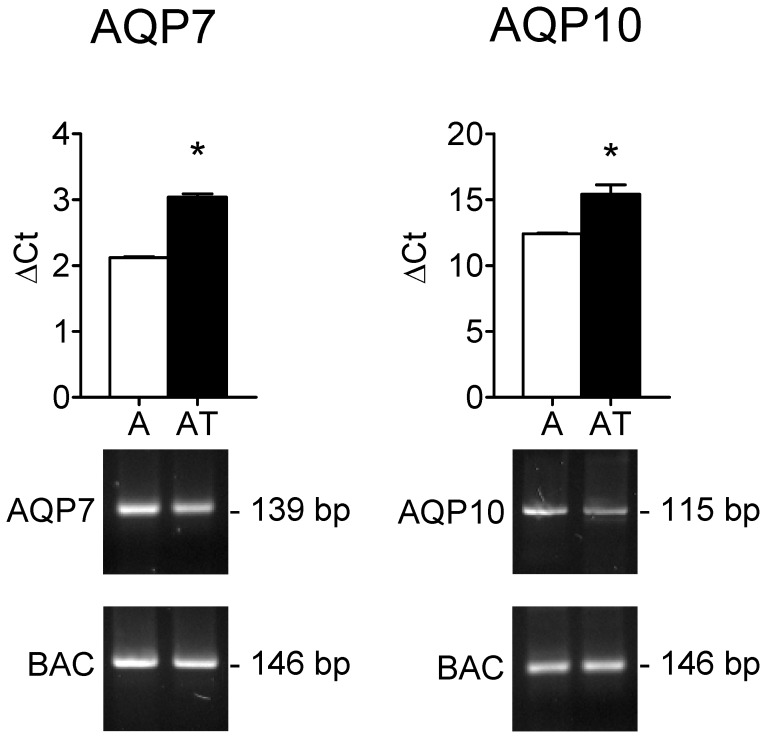
Aquaporin-7 (AQP7) and -10 (AQP10) mRNA expression in human subcutaneous adipose tissue (AT) and in isolated adipocytes (A). Upper panels, mRNA levels were measured by real-time RT-PCR relative to the β-actin internal standard (see Materials and Methods section) and the values obtained were reported as ΔCt. Bars represent the mean ± SEM of at least 4 different experiments each from different RNA extracts. *P<0.05 versus A (Student’s *t* test). Lower panels, Gel electrophoresis of the PCR products. Specific PCR products for AQP7 (139 bp band), AQP10 (115 bp band) and β-actin (146 bp) were observed in both AT and A.

First we explored by qRT-PCR the expression of AQP7 and 10 mRNA in human subcutaneous adipose tissue, and in isolated adipocytes as well. AQP7 and 10 transcripts were expressed in subcutaneous adipose tissue (Fig. 1), while AQP6 transcript, used as negative control, was absent (not shown). The results of agarose gel electrophoresis of representative PCR reaction products are also shown in Fig. 1.

Single bands of the expected size of cDNA fragments were amplified (139 and 115 bp for AQP7 and AQP10, respectively) and the specificity of the cDNAs amplified by RT-PCR was confirmed by sequencing the PCR products. Negative controls of RT-PCR experiments were always performed by omitting the reverse transcriptase (not shown).

Since AQP7 has been recently localized in the capillary endothelium of white adipose tissue in rats, and, surprisingly, not in adipocytes [15], we studied AQP7 and 10 expression also in isolated adipocytes to exclude endothelial mRNA contamination. The results confirmed that isolated adipocytes expressed AQP7 and 10 transcript (Fig. 1). No differences were observed in aquaporin expression between adipose tissue and isolated adipocytes. Moreover, AQP7 mRNA expression results significantly higher than that of AQP10 in both adipose tissue and isolated adipocytes, with high ΔCt values reflecting low mRNA expression levels (Fig. 1).

### Immunoblotting of AQP7 and 10 in Human Adipose Tissue Plasma Membranes

**Figure 2:**
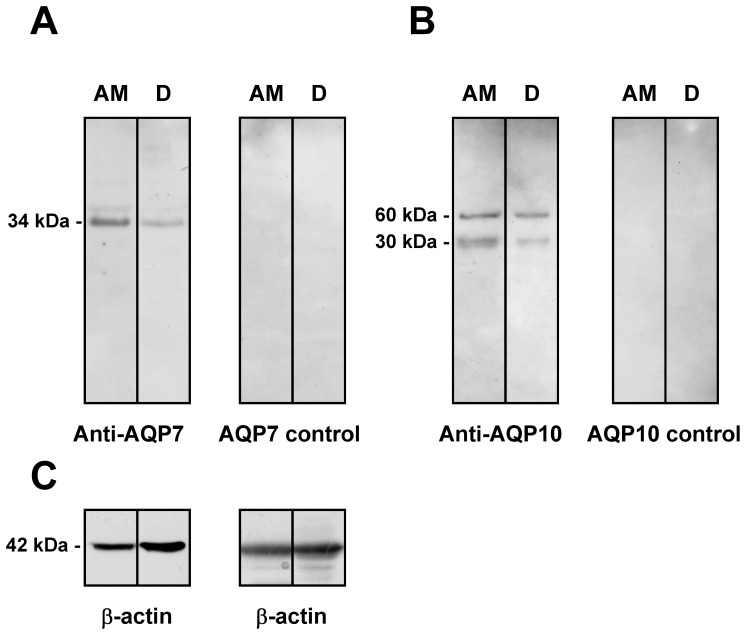
Aquaporin-7 (A), and -10 (B) protein expression in human adipocytes plasma membranes. Blots representative of four were shown. Lanes were loaded with 35 µg of proteins, probed with anti-AQP7 rabbit polyclonal antibody (A left) and processed as described in Materials and Methods. The same blots were stripped and re-probed with anti-AQP10 rabbit polyclonal antibody (B left), and anti-β-actin antibody as housekeeping (C). A major band of about 34 kDa and two bands of about 30 kDa (monomer) and 60 kDa (dimer) were observed when the blots were probed with anti-AQP7 and anti-AQP10 antibodies respectively. No bands were detected when preadsorbed anti-aquaporin-7 or -10 antibodies were used (A and B right). AM, adipocytes plasma membranes. D, duodenal crude homogenate.

Plasma membrane from human subcutaneous adipose tissue and from duodenal biopsy (positive control) were analyzed by immunoblotting with affinity-purified antibodies against human AQP7 and 10. The results showed that AQP7 and 10 proteins were expressed in both tissues plasma membranes (Fig. 2A and B, respectively). A major band at 34 kDa was observed in immunoblots probed with anti-AQP 7 antibody (Fig. 2A left). Immunoblots of AQP10 protein showed a major band at approximately 30 kDa, that was compatible with previous findings [21], [22], and a band of approximately 60 kDa, probably representing the dimer form (Fig. 2B left). The specificity of the reactions was confirmed by preadsorption experiments for both AQP7 and 10. In both cases the protein bands were absent (Fig. 2A and B right). The expression of the housekeeping gene β-actin in both tissue homogenates was also shown (Fig. 2C).

### Immunolocalization of AQP7 and 10 Protein in Human Adipose Tissue

**Figure 3:**
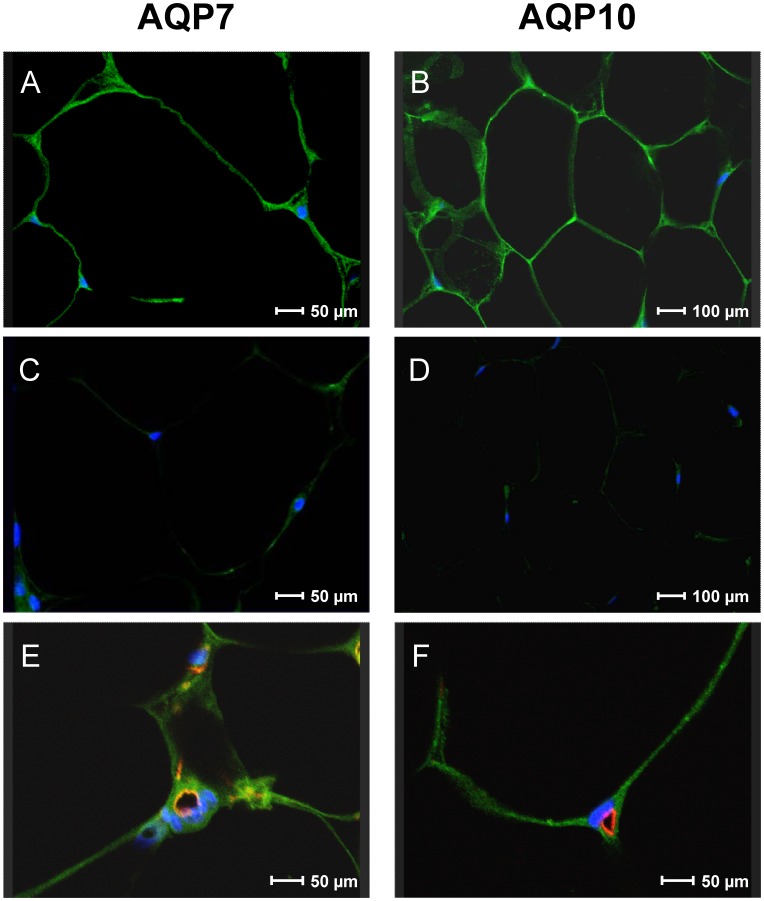
Representative immunofluorescence confocal microscopical images of aquaporin-7 and -10 localization in human subcutaneous adipose tissue. Green labeling indicates the presence of aquaporin-7 (A) and -10 (B) at the plasma membrane and at the cytoplasm of the human adipocytes. No or faint staining was observed when anti-aquaporin-7 and anti-aquaporin-10 preadsorbed antibodies were used (C-D). Nuclei were counterstained by DAPI (blue). Colocalization of aquaporin-7 or -10 and the endothelial cell marker CD34 in human subcutaneous adipose tissue (E-F) was performed as detailed in “Materials and methods”. Green labeling indicated the presence of aquaporin-7 (E) or -10 (F), red labeling the vessels, while nuclei were counterstained by DAPI (blue). Merged images showed strong colocalization signal of aquaporin-7 and CD34 (yellow labelling) in the capillary, even though aquaporin-7 was also expressed in the adipocytes (E). On the contrary, aquaporin 10 and CD34 did not colocalized (F).

The cellular and sub-cellular localization of AQP7 and 10 protein in human subcutaneous adipose tissue was investigated by immunofluorescence. As shown in Fig. 3, the anti-AQP7 and anti-AQP10 antibodies strongly labeled the human adipose tissue both to the plasma membrane and to a thin rim of cytoplasm of adipocytes (Fig. 3A and B). Negative controls (with depleted anti-AQP7 and 10) gave a faint or negligible signal (Fig. 3C and D).

To exactly define the localization of the two aquaglyceroporins in adipose tissue, we investigated the co-localization of AQP7 or AQP10 with CD34, an endothelial cell marker. Double label immunofluorescence showed that AQP7 and CD34 proteins were highly co-localized in endothelium of adipose tissue, but an intense AQP7 labeling of adipocytes was also observed (Fig. 3E). On the contrary AQP10 was expressed only in adipocytes, as per the resulting absence of yellow fluorescence in merged images of CD34 (red) and AQP10 (green) (Fig. 3F). The different labeling patterns of AQP10 and CD34 confirmed the different localization of these proteins in human white adipose tissue.

**Figure 4:**
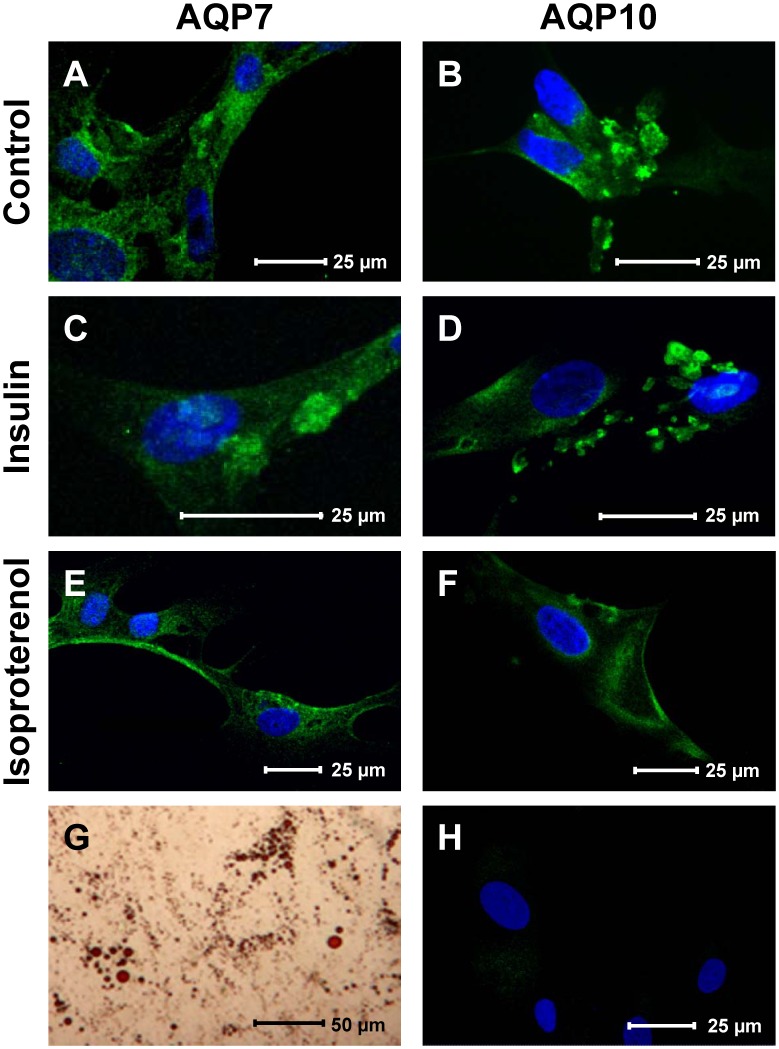
Effect of insulin and isoproterenol on subcellular localization of aquaporin-7 (AQP7) and -10 (AQP10) in human cultured differentiated adipocytes. The aquaporin localization was detected in basal conditions (control: A, B), after insulin (C, D) or isoproterenol (E, F) stimulation. Control adipocytes show an intracellular AQP7 and AQP10 green labeling, particularly evident around small lipid droplets. Green labeling indicated the presence of AQP-7-10, while nuclei were counterstained by DAPI (blue). Insulin treatment increased the AQP7 and AQP10 staining around the lipid droplets while that of isoproterenol reduced the lipid droplets labeling, greatly increasing the plasma membrane staining. Negative controls gave a faint or negligible signal (H). Accumulation of lipid droplets in adipocytes was demonstrated by Oil Red O staining (G).

To study the possible regulation of AQP10 translocation by insulin and or β-agonist treatment we evaluated the AQP7 and AQP10 immunolocalization in differentiated adipocytes treated as described [11]. Differentiation of human mesenchymal stem cells (ASCs) into adipocytes was evaluated by Oil Red O staining to detect intracellular lipid accumulation (Fig. 4G).

In control serum-starved adipocytes both AQP7 and AQP10 labeling was intracellular and particularly evident around small lipid droplets (Fig. 4A and B). In few control adipocytes the AQP10 labeling was mainly on the plasma membrane. Insulin treatment increased the AQP7 and AQP10 staining around the lipid droplets (Fig. 4C and D; Movie S1). Conversely, the isoproterenol treatment reduced the lipid droplets labeling of both water channels increasing the plasma membrane staining (Fig. 4 E and F) suggestive of a membrane trafficking mechanism for the AQP10 similar to that previously demonstrated for AQP7 [11]. Negative controls gave a faint or negligible signal (Fig. 4H).

### Water and Glycerol Permeability of Isolated Adipocytes and of ***adipocyte*** Plasma Membrane Vesicles

**Figure 5:**
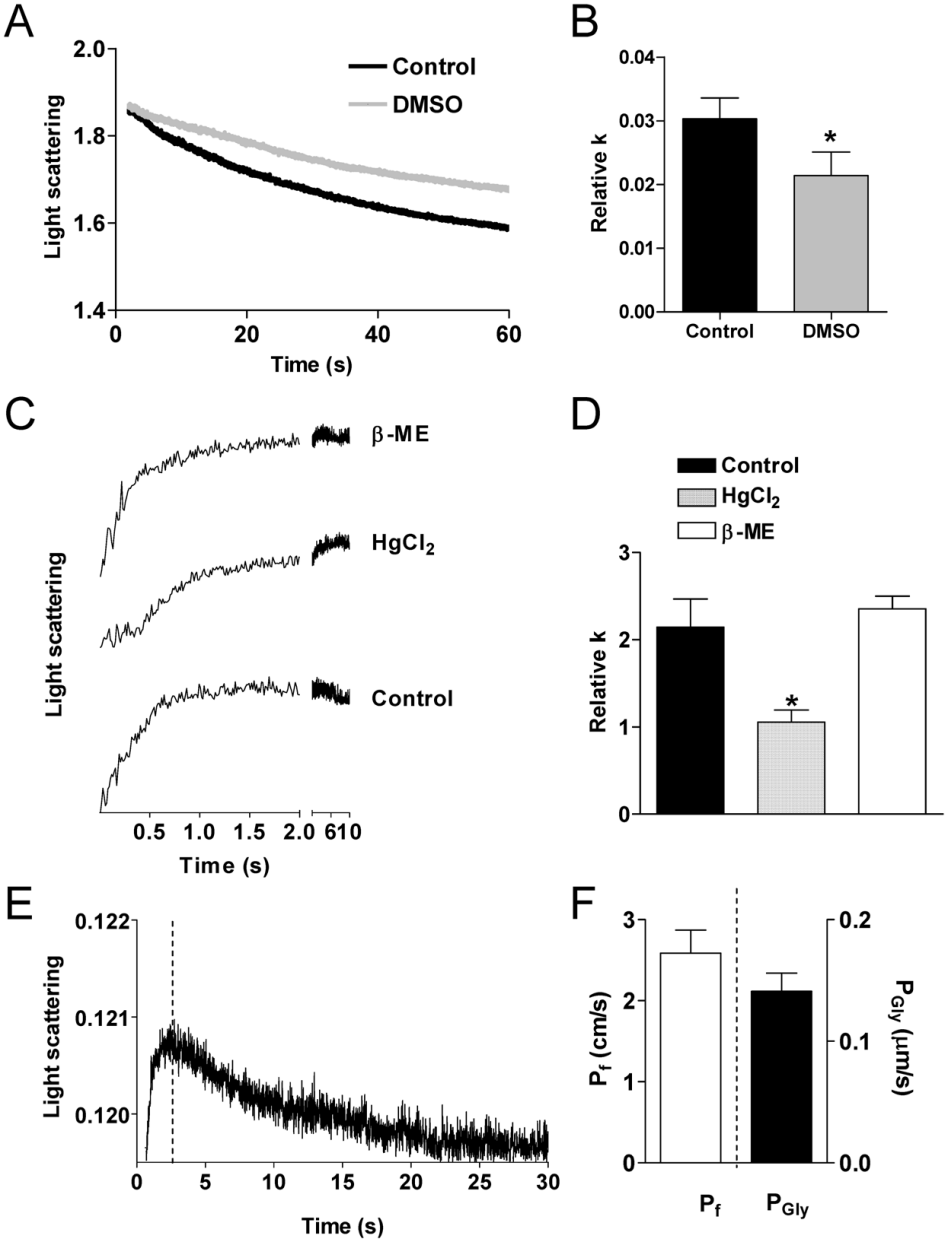
Water and glycerol permeability of human adipocyte plasma membrane vesicles. (A) Representative light scattering curves were obtained by exposing the isolated adipocytes to a 150 mOsm osmotic gradient in two different conditions: normal untreated cells (Control) and cells treated for 15 min with 0.5 M DMSO (DMSO). (B) Bars represent water permeability of isolated adipocytes, expressed as relative k. Values are means ± SEM of at least 15 single shots for each of five different adipocyte preparations. *, P<0.05 vs Control (Student *t* test for pair data). (C) Representative light scattering curves were obtained by exposing the adipocytes plasma membrane vesicles to a 150 mOsm osmotic gradient in three different conditions: normal untreated vesicles (Control), vesicles treated for 10 min with 1 mM HgCl_2_, vesicles treated with 1 mM HgCl_2_ followed by 15 min treatment with 15 mM β-mercaptoethanol (β-ME). (D) Bars represent water permeability of adipocyte plasma membrane vesicles, expressed as relative k. Values are means ± SEM of at least 8 single shots for each of five different preparations. *, P<0.05 vs Control and β-ME (repeated measure ANOVA, followed by Newman-Keuls’s *Q* test). (E) Representative light scattering curves were obtained by exposing the adipocytes plasma membrane vesicles to a 150 mM inwardly directed gradient of glycerol. The initial increase in light scattering results from osmotic water efflux caused by vesicle shrinkage (before the dashed line), while the subsequent slower decrease is caused by glycerol entry (after the dashed line). (F) Water (Pf) and glycerol (Pgly) permeability coefficients were calculated as described in Materials and Methods.

Isolated adipocytes (mean *cell diameter of 60±2.7 µm) exposed to a hypotonic buffer behaved* as functional osmometers showing a sudden swelling (Fig. 5A). The decrease in scattered light intensity could be fitted by a one phase exponential decay equation and the initial rate constants k obtained. Adipocytes pretreated with the water channel inhibitor DMSO [25], [26], exhibited a significant decrease in water transport when exposed to a hypotonic environment (Fig. 5B; k value reduced by about 30% P<0.05 Student’s t test for pair data).

Electron microscopy and morphometric analysis showed that adipocyte plasma membrane vesicles were consistently spherical (not shown), with the shape factor of about a unit, and mean diameter of about 72.3±5.9 nm. Vesicle preparations behaved as functional osmometers showing rapid shrinkage in response to a hyperosmotic solution recorded as an increase in light scattering (Fig. 5C). In control experiments, no signal change was observed in the absence of an osmotic gradient (not shown). Involvement of AQPs in vesicles shrinkage was confirmed by the observation that the pretreatment of the vesicles with AQP water channel inhibitor Hg^2+^ significantly reduced about 50% of the water transport values. Hg^2+^ inhibition was completely reversed by treatment with β-mercaptoethanol (P<0.05, ANOVA followed by Newman-Keuls’s *Q* test; Fig. 5C and D).

Glycerol permeability was measured by recording the light scattering changes after exposing the adipocyte plasma membrane vesicles to a 150 mM inwardly directed gradient of glycerol.

as described by Yang and coworkers [27]. Fig. 5E shows a representative biphasic light scattering curve from which it was possible to calculate the water (Pf) and glycerol (Pgly) permeability coefficients as described in Materials and Methods (Fig. 5F). The initial increase in light scattering results from osmotic water efflux caused by vesicle shrinkage (before the dashed line), while the subsequent slower decrease is caused by glycerol entry (after the dashed line) (Fig. 5E).

### Gene Silencing

**Figure 6:**
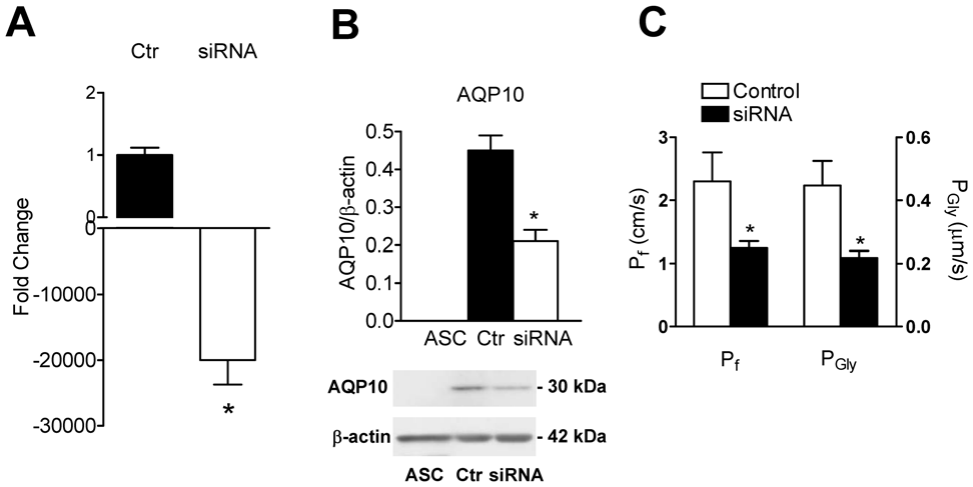
Aquaporin-10 (AQP10) silencing in human differentiated adipocytes. AQP10 short interfering RNA (siRNA) and scrambled siRNA (Ctr) were transfected in differentiated adipocytes as described in Materials and methods. A, AQP10 mRNA levels were measured by real-time RT-PCR relative to the β-actin internal standard and the values obtained were reported as fold change (see Materials and Methods section). Bars represent the mean ± SEM of at least 4 different experiments each from different RNA extracts. AQP10 transcript was reduced in silenced differentiated adipocytes compared to controls; *P<0.006 versus Ctr (Student’s *t* test). B, Western blot and densitometry demonstrate that undifferentiated adipose stem cells (ASC) had no AQP10 expression, while AQP10 protein was reduced in silenced differentiated adipocytes (siRNA) compared to controls (scrambled; Ctr)(*, P<0.003; Student’s t test). Blots representative of three were shown (B, lower pannel). The same blots were stripped and re-probed with anti-β-actin antibody. Bands of the expected molecular weights were shown and acquired with the Image Master VDS (GE Healthcare Life Sciences, Italy). Densitometric analysis of the bands was performed by Total Lab V 1.11 computer program (GE Healthcare Life Sciences, Italy) and the results were normalized to the corresponding β-actin (B, upper panel). C, Water (Pf) and glycerol (Pgly) permeability coefficients were calculated as described in Materials and Methods. Both Pf and Pgly were significantly reduced in siRNA compared to controls by 46% and 51%, respectively (*, P<0.002; Student’s t test).

Short interfering (siRNA) targeting human AQP10 was used to determine the contribution of AQP10 in mediating glycerol transport in human adipocytes. The effectiveness in silencing was preliminarily determined by qRT-PCR and by immunoblotting. The results showed that AQP10 transcript was significantly reduced and sometimes completely removed (Fig. 6A). Immunoblotting experiments were performed to confirm the AQP10 protein reduction in knocked down-cells. The results demonstrated that undifferentiated adipose stem cells (ASC) had no AQP10 expression, while AQP10 protein was knocked-down of about 50% in silenced differentiated adipocytes compared to differentiated adipocytes (controls) (Fig. 6B). On the contrary, AQP7 mRNA and protein expression is unmodified in AQP10 KO adipocytes (Figure S1). Successively, water and glycerol permeability was measured by recording the light scattering changes after exposing the differentiated adipocytes plasma membrane vesicles to an inwardly directed glycerol gradient. Both Pf and Pgly were significantly reduced in silenced adipocytes compared to controls by 46% and 51%, respectively (Fig. 6C). This result directly demonstrates the contribution of the aquaporin-10 in the overall glycerol and osmotic water permeability of differentiated adipocytes.

## Discussion

The study of aquaglyceroporins expression in human subcutaneous adipose tissue will undoubtedly provide important information in understanding glycerol transport mechanisms through the adipocytes, a crucial step in the maintenance of normal adiposity. In this specific contest, we have demonstrated using different methods (RT-PCR, immunoblotting, fluorescence microscopy, and gene silencing) that AQP10 is present in human adipocytes and can be considered an alternative pathway for glycerol efflux in addition to the previously demonstrated aquaglyceroporins.

AQP7 and 10 belong to the aquaporin family, and based on their functional characteristics, to the sub-group of the aquaglyceroporins [5]. Thus, their permeability was confined not only to water but also to glycerol, urea and other small solutes.

AQP7 is present in various tissues, such as gastrointestinal tract, kidney, skeletal muscle, inner ear and male reproductive system, but the adipose tissue represents the major site of AQP7 expression in humans and rodents where it reportedly promotes glycerol exit [5]. Nevertheless the localization of AQP7 in adipocyte plasma membrane has been recently questioned. Nielsen’s group developed an AQP7-KO mice line to study accurately the tissue localization of AQP7 and its role in glycerol metabolism [15]. Surprisingly, AQP7 protein was localized in the capillary endothelium of adipose tissue but not in adipocyte membranes.

Even if obtained in mice, the localization of AQP7 in the capillary endothelium rather than in adipocyte membranes represents a Copernican revolution in the field that opens new questions and widens the horizons.

In this direction we consider also the expression and localization of AQP7 in human adipose tissue. We found that AQP7 transcript was present not only in adipose tissue, that obviously possesses capillaries, but also in isolated and in cultured differentiated adipocytes. At protein level AQP7 expression was demonstrated by immunoblotting and using confocal immunofluorescence. Results clearly show a labeling either in the capillary endothelium or in the adipocyte membranes. Colocalization of AQP7 with CD34, well known marker of endothelial cell, confirms that AQP7 is, at least in part, associated with the plasma membrane of the small vessel of mouse adipose tissue [15]. To be more convinced on AQP7 membrane localization we verified its expression and localization in cultured differentiated adipocytes. The labeling observed seems to support an AQP7 expression in adipocyte membranes in addition to the endothelial cell.

The core of this study focused however on the identification of an alternative pathway in human adipocytes plasma membrane that works together with AQP7 in glycerol outflow to adipose interstitium. The results presented here show that AQP10 is expressed in human white adipose tissue and is localized in the plasma membrane of fat cells. AQP10 mRNA was demonstrated in native subcutaneous adipose tissue, in isolated and in cultured differentiated adipocytes, even though in different amount. Immunoblotting of crude membrane confirmed also at protein level the presence of AQP10 in human adipose tissue with bands of the expected size for the monomeric and dimeric forms, as previously reported [21]. Moreover, the bands were similar to those of duodenal membrane homogenates used as positive control (Fig. 2B left) [22]. Differently from AQP7, AQP10 appears localized exclusively at the plasma membrane domain of adipocytes (Fig. 3F). Moreover, co-localization experiments performed with anti-CD34 antibody, excluded the AQP10 presence in the plasma membrane of small vessels of adipose tissue. Interestingly, a short AQP10 variant has been recently found in the capillary endothelium of human small intestine [28]. In this regard, though the expression of AQP10 has been demonstrated, until now, almost exclusively in the gastrointestinal tract, a clear-cut protein localization has not unanimously accepted. Unlike initially observed by *in situ* hybridization and immunohistochemistry in villous superficial epithelial cells, a later study found two AQP10 forms expressed in granules of entero-endocrine cells and, as above stated, in the small intestinal capillary endothelium [5], [22], [23], [28]–[30].

Functionally, aquaglyceroporins existence in human adipocyte plasma membranes has been also demonstrated by measuring osmotic water and glycerol permeability of isolated adipocytes and adipocyte plasma membranes preparations. These preparations behaved as functional osmometers sensitive to DMSO or Hg^2+^ treatment. Nevertheless, this result was not able to distinguish the single contribution of different aquaglyceroporins to the overall water and glycerol permeability of adipocytes since all these AQPs have been shown to be sensitive to mercurial compounds [2], [21], [23].

The expression and regulation of AQP7 was extensively studied in human adipose tissue since its primary function as a glycerol- rather then as a water-channel. Reduced glycerol outflow from the adipocytes has been suggested to be a crucial step that leads to adipocyte hypertrophy, increased adiposity until obesity onset. [12], [13], [31].This theory derives from exciting results obtained with AQP7-KO mice independently generated by two research groups that displayed an increase in body weight and fat accumulation in adulthood [12], [13]. These findings were confirmed also by in *in vitro* results with AQP7-knockdown 3T3-L1 adipocytes [13]. The results are in disagreement with those obtained using other AQP7-null mice, independently generated by Matsumura et al. [16] and by Nielsen’s group [15] that exhibited normal body weight, growth curves and adipose masses, normal lipolytic activity and normal glycerol plasma concentration. In humans, AQP7 gene expression was found to be down-regulated in the adipose tissue of obese vs. lean subjects [17], [18], but unaltered in type 2 diabetes subjects [17]. A common polymorphism in the promoter region of human AQP7 gene (A-953G SNP) was found to reduce AQP7 expression in adipose tissue thus increasing the risk of obesity and eventually type 2 diabetes [19]. However, differently from studies on AQP7-null mice, a clear correlation between AQP7 expression and obesity failed to be observed. Indeed, human subjects homozygous for G264V mutation encoding for a non- functional AQP7 protein have normal adiposity and normal plasma glycerol concentration in basal conditions [17], [20]. This has strengthened the hypothesis of alternative glycerol channels in adipocytes [17]. The results reported here can clarify the different findings observed between humans and mice: i.e., the human adipose tissue expresses AQP10 that in mice has been demonstrated to be a pseudogene [24]. Results here presented demonstrate that AQP10 is regulated by lipogenic (insulin) and lipolytic (isoproterenol) stimuli thus participating to the control of fat accumulation. This regulation is similar to that described for AQP7 in 3T3-L1 adipocytes [11]. These authors have also demonstrated in human subcutaneous and omental adipose tissue the presence of AQP3 and 9, with the first favouring the glycerol exit after lipolytic stimuli and the latter being constitutively present in the plasma membrane and involved in glycerol entry to lower hyperglycaemia.

In conclusion, the present study demonstrates on one hand the expression of AQP7 in human adipocyte plasma membrane and on the other hand the presence of an alternative glycerol channel, AQP10, that working with AQP3 and AQP7 ensures glycerol exit from adipocyte, thus protecting humans from obesity.

However, further investigation is required to clarify AQP10 gene expression regulation in obese and in diabetic subjects in respect to that of AQP7.

## Materials and Methods

### Subjects

Human subcutaneous fat tissues were obtained from the subjects (age 60–76 years, 80% females) under surgical interventions, after an overnight fasting. The body mass index of the donors ranged from 21 and 31 kg/m^2^ (25.32±0.96, mean ± S.E.M.). None of the subjects suffered from known metabolic or malignant diseases nor were they taking medications known to alter the adipose tissue metabolism. The procedures followed were approved by the Institutional Review Board at “Istituto di Ricovero e Cura a Carattere Scientifico Policlinico San Matteo Foundation” in Pavia, and in accordance with the Helsinki Declaration of 1975 as revised in 2008. Each patient gave written informed consent for participation in the study.

### Adipocytes Isolation and Culture

Excised adipose tissue samples (1.3–1.5 g w.t.) were minced in pieces of ∼10 mg in weight and incubated for 55–60 min in Erlenmeyer plastic flasks with 4 ml of a medium containing DMEM F-12 HAM (Sigma), 25 mM HEPES, 40 mg/ml BSA, 2 mg/ml collagenase type II, pH 7.4 at 37°C in a thermostatic shaker, as previously described [32]. Adipocytes were then filtered through 180 µm mesh filters and washed three times by diluting with HANKS, 20 mM HEPES, 1% BSA, pH 7.4 and centrifuging at room temperature for 1 min at 400 *g*. Adipocytes were stored in ice until used.

Cultured human adipocytes were obtained after isolation of mesenchymal stem cells (ASCs) followed by their differentiation. ASCs were obtained following Gastaldi et al. [33]. Subcutaneous adipose tissue was digested by collagenase treatment as above described, diluted with ice-cold DMEM F-12 HAM and centrifuged at 400 *g* for 10 min at 4°C. The ASCs were resuspended in a RBC lysis buffer (155 mM NH_4_Cl,10 mM KHCO_3_, 0.1 mM EDTA, pH 7.3) for 10 min and centrifuged again. Cells were resuspended in DMEM F-12 HAM supplemented with 10% FBS (Sigma), seeded onto polystyrene tissue culture dishes (35 mm diameter) and maintained at 37°C in a humidified atmosphere of 5% CO_2_, 95% air. The adherent cells were trypsinized and 1×10^5^ ASCs per 100 mm^2^ tissue culture plate were seeded in flasks. Afeter three passages ASCs were grown to confluence and then cultured for the first 3 days with a chemically defined serum-free preadipocyte differentiation medium containing 8 µg/ml d-biotin, 0.5 µg/ml bovine insulin, 400 ng/ml dexamethasone, 44 µg/ml isobutyl-methylxanthine, 9 ng/ml L-thyroxine, 3 µg/ml ciglitazone (Promocell; Cat. No.: C-27437). Cells were then fed for 12–14 days with adipocyte basal medium containing 3% FCS, 8 µg/ml d-biotin, 0.5 µg/ml bovine insulin and 400 ng/ml dexamethasone (Promocell; Cat.-No.: C-27439). Medium was changed every 48–72 hours.

All the media were supplemented with 100 U/ml penicillin, 100 µg/ml streptomycin, and 0.25 µg/ml amphotericin B final concentration.

### Cell Size

An aliquot of freshly isolated adipocytes was stained with methylene blue (0.02 g/100 ml PBS), examined by light microscopy using an Olympus BX41 and the digital images acquired with the Nikon DS-Fi1 digital camera using Nis Element F Imaging Software. Cell diameters were measured by the Scion Image Beta 4.02 computer program (Scion Corporation).

### Plasma Membrane Vesicles Isolation

Adipocyte plasma membrane vesicles were prepared according to Oka et al. [34]. Briefly, 2.5–8 g freshly excised adipose tissue was homogenized at a 7 ml/1 g ratio in an ice-cold solution containing 250 mM sucrose, 1 mM EDTA, 10 mM Tris-HCl, pH 7.4, using a Potter-Elvehjem homogenizer (15 strokes). The homogenate was centrifuged at 3000 *g* for 15 min at 4°C, the superficial solidified fat was eliminated, and the infranatant was centrifuged again for 15 min at 12.000g. The resulting pellet consists of adipocyte plasma membrane vesicles. Morphologic appearance of the vesicles was assessed by a conventional electron microscopy technique and the diameters measured by the Scion Image Beta 4.02 computer program (Scion Corporation).

### RNA Isolation and Real Time RT-PCR (qRT-PCR)

Total RNA was extracted from human subcutaneous adipose tissue, isolated and cultured adipocytes using QIAzol Lysis Reagent (Qiagen SpA, Milan, Italy). Reverse transcription was performed according to Laforenza et al. [29].

qPCR was carried out as previously described [29] in triplicate using 1 µl cDNA and specific primers: AQP7, Hs_AQP7_1_SG QuantiTect Primer Assay QT00067592 (Qiagen) and AQP10, Hs_AQP10_1_SG QuantiTect Primer Assay QT00043393 (Qiagen). Briefly, GoTaq qPCR Mastermix (Promega, Italy) was used according to the manufacturer instruction and qPCR performed using Rotor Gene 6000 (Corbett). The conditions were as follows: initial denaturation at 95°C for 5 min; 40 cycles of denaturation at 95°C for 30 s; annealing at 58°C for 30 s, and elongation at 72°C for 40 s. The qPCR reactions were normalized using β-actin as housekeeping gene (Hs_Actb_1_SG, QuantiTect Primer Assay QT00095431, Qiagen, Italy). Melting curves were generated to detect the melting temperatures of specific products immediately after the PCR run. The triplicate threshold cycles (Ct) values for each sample were averaged resulting in mean Ct values for both the gene of interest and the housekeeping gene β-actin. The gene Ct values were then normalized to the housekeeping gene by taking the difference: ΔCt = Ct[gene] − Ct[β-actin], with high ΔCt values reflecting low mRNA expression levels. In silencing experiments results were expressed as fold change:
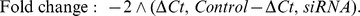



PCR products were also separated with agarose gel electrophoresis, stained with ethidium bromide, and acquired with the Image Master VDS (GE Healthcare Life Sciences, Italy). The molecular weight of the PCR products was compared to the DNA molecular weight marker VIII (Roche Molecular Biochemicals, Italy).

### Immunoblotting

Plasma membrane vesicles from human subcutaneous adipose tissue and from duodenal biopsy were treated as previously described [35]. 35 µg of solubilised proteins were subjected to 12.5% SDS-polyacrilamide gel electrophoresis and transferred to the Hybond-P PVDF Membrane (GE Healthcare, Italy) by electroelution. The membranes were treated as previously described [36] and then incubated overnight with anti-AQP7 rabbit polyclonal IgG (sc-28625), anti-AQP10 rabbit polyclonal IgG affinity pure (Alpha Diagnostics Intl. Inc. or ABgent Inc., U.S.A.), diluted 1∶500 and 1∶2000 in the blocking solution, respectively. The membranes were washed and incubated for 1 h with peroxidase-conjugated goat anti-rabbit IgG (GE Healthcare, Italy), diluted 1∶100,000 in blocking solution. The bands were detected with ECL™ Advance western blotting detection system (GE Healthcare, Italy). Control experiments were performed by using the antibody preadsorbed with a 20-fold molar excess of the immunizing peptide for AQP7 and AQP10. Prestained molecular weight markers (SDS7B2, Sigma, Italy) were used to estimate the molecular weight of the bands. Blots were stripped [37] and re-probed with anti β-actin rabbit antibody as loading control (Rockland Immunochemicals for Research, U.S.A.; code, 600-401-886). The antibody was diluted 1∶2000 in blocking solution.

### Immunofluorescence

Immunolocalization of AQP7 and 10 was evaluated both in human adipose tissue and in cultured adipocytes. Human subcutaneous fat tissue was fixed with acetate buffered 4% formalin for 24 h and embedded in paraffin. Rehydrated paraffin sections (10 µm) were washed once with PBS and then blocked with 3% BSA in PBS for 30 min at room temperature. Slides were incubated for 2 h at room temperature with affinity pure primary antibodies (see Immunoblotting sections) diluted 1∶2000 in antibody diluent (Dako). After three 5 min washes with PBS, slides were incubated for 30 min at room temperature with fluorescent secondary antibody (goat anti-rabbit antibody AlexaFluor 488, Molecular Probes).

In double labeling experiments sections were incubated for 2 h at room temperature with rabbit anti-AQP7 or AQP10 and monoclonal mouse anti-CD34, endothelial cell marker, (clone QBEnd/10; YLEM, Avezzano, Italy). After being washed, slides were stained simultaneously with two secondary antibodies goat anti-rabbit AlexaFluor 488 and donkey anti-mouse AlexaFluor 594 (1∶1000 dilution; Molecular Probes) for 30 min at room temperature.

Slides were then washed 3×5 min with PBS, mounted in ProLong® Gold antifade reagent with DAPI (Molecular Probes) and examined with a TCS SP2 LEICA confocal microscopy system equipped with a LEICA DM IRBE inverted microscope. Control experiments were performed simultaneously using anti-AQP7 and anti-AQP10 antibodies preadsorbed with immunizing peptides or using non-immune serum.

In regulation experiments, differentiated adipocytes were treated as described by Rodriguez et al. [11]. Briefly, adipocytes grown on 24×24 mm glass coverslips were serum-starved for 2 h (control cells) and then stimulated for 30 min with insulin (100 nmol/L) or isoproterenol (10 µmol/L). Cells were fixed with 3% paraformaldehyde in PBS for 20 min at room temperature [38] and then washed four times with PBS. After removing the medium and washing the cells with PBS, the cells were fixed with 10% formalin.

The effective differentiation into adipocytes was demonstrated by accumulation of neutral lipids, detected by staining the cells in a solution of 0.5% Oil Red-O at room temperature for at least 60 min (Fig. 4G).

### Water and Glycerol Permeability Measurements

Osmotic water permeability of isolated adipocytes and adipocyte plasma membrane vesicles was measured by stopped-flow light scattering method [39] as previously described [40]. The initial rate constant of cells and vesicles volume changes (k) was obtained by fitting the time course of light scattering with a one phase exponential decay or a single exponential equation for isolated adipocytes and plasma membrane vesicles, respectively (GraphPad Prism 4.00, 2003). The water permeability coefficient, Pf, was calculated as previously described by Wiener et al. [41], from the following equation:
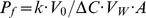
where *ΔC* is the osmotic gradient, *V_w_* the molar water volume, *V_0_* the cell volume and *A* the cell surface area. *V_o_* and *A* were obtained as above described. Water transport in isolated adipocytes was evaluated in a) normal untreated cells; b) cells treated for 15 min with 0.5 M DMSO exposed to hypoosmotic solution. Water transport in adipocyte vesicles was evaluated in: a) normal untreated vesicles; b) vesicles treated for 10 min with 1 mM HgCl_2_; c) vesicles treated for 10 min with HgCl_2_ followed by 15 min treatment with 15 mM β-mercaptoethanol. Vesicles were first incubated in hypoosmotic solution containing 100 mM D-mannitol and Tris/Hepes 5 mM, pH 7.4, and then were exposed to hypertonic solution containing 400 mM D-mannitol and Tris/Hepes 5 mM, pH 7.4.

Glycerol permeability of isolated adipocyte plasma membranes was measured by stopped-flow light scattering method [27]. Vesicles were subjected to a 150 mM inwardly directed glycerol gradient and the glycerol permeability (P_gly_) was calculated using the following equation:



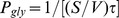
where S is the cell surface area, V the cell volume, and τ is the exponential time constant fitted to the vesicle swelling phase of the light scattering time course corresponding to glycerol entry [27].

### Gene Silencing

Short interfering RNA (siRNA) targeting human AQP10 was purchased by Sigma (esiRNA human AQP10 EHU065251). Briefly, the medium was removed from differentiated adipocytes and the cells were added with Opti-MEM I reduced serum medium without antibiotics (Opti-MEM) (Life technologies, U.S.A.). siRNA (75 nM final concentration) were diluted in Opti-MEM and mixed with Lipofectamine™ RNAiMAX transfection reagent (Invitrogen) pre-diluted in Opti-MEM according to the manufacturer’s instructions. After 20 min incubation at room temperature, the mix was added to the cells and incubated at 37°C for 5 h. The transfection mixes were then completely removed and fresh culture media were added. Scrambled siRNA were used as negative control. KO-cells were used 48 hours after transfection. The effectiveness in silencing was determined by real time RT-PCR and by immunoblotting.

### Protein Content

The protein content was determined with the Bradford method [42] using bovine serum albumin as standard.

### Statistics

All data were expressed as means ± S.E.M. The significance of the differences of the means were evaluated by using one-way ANOVA followed by Newman-Keuls’s *Q* test, or Student’s *t* test. All statistical tests were carried out with GraphPad Prism 4.00, 2003.

## Supporting Information

Figure S1 **Aquaporin-7 (AQP7) expression in aquaporin-10 (AQP10) silenced human differentiated adipocytes.** AQP10 short interfering RNA (siRNA) and scrambled siRNA (Ctr) were transfected in differentiated adipocytes as described in Materials and methods. A, AQP7 mRNA levels were measured by real-time RT-PCR relative to the β-actin internal standard and the values obtained were reported as fold change (see Materials and Methods section). Bars represent the mean ± SEM of at least 4 different experiments each from different RNA extracts. AQP7 transcript was unmodified in silenced differentiated adipocytes (siRNA) compared to controls (scrambled; Ctr) (P = 0.186; Student’s *t* test). B, Western blot and densitometry demonstrate that also AQP7 protein was unmodified (P = 0.872; Student’s t test). Blots representative of three were shown (B, lower pannel). The same blots were stripped and re-probed with anti-β-actin antibody. Bands of the expected molecular weights were shown and acquired with the Image Master VDS (GE Healthcare Life Sciences, Italy). Densitometric analysis of the bands was performed by Total Lab V 1.11 computer program (GE Healthcare Life Sciences, Italy) and the results were normalized to the corresponding β-actin (B, upper panel).
(TIF)

Movie S1 **Subcellular localization of aquaporin-10 (AQP10) in human cultured differentiated adipocytes after insulin stimulation.** Insulin treatment increased the AQP10 staining around the lipid droplets. The movie shows a representative AQP10 labeling (green) in a confocal 3D reconstruction. Nucleus was counterstained with DAPI (blue). (see also Figure 4D).
(AVI)
