# Supplementary figures and images for: Aquaporin-10 Represents an Alternative Pathway for Glycerol Efflux from Human Adipocytes
Source: PLoS One. 2013 Jan 29;8(1):e54474. doi: 10.1371/journal.pone.0054474 (PMC3558521; doi:10.1371/journal.pone.0054474)

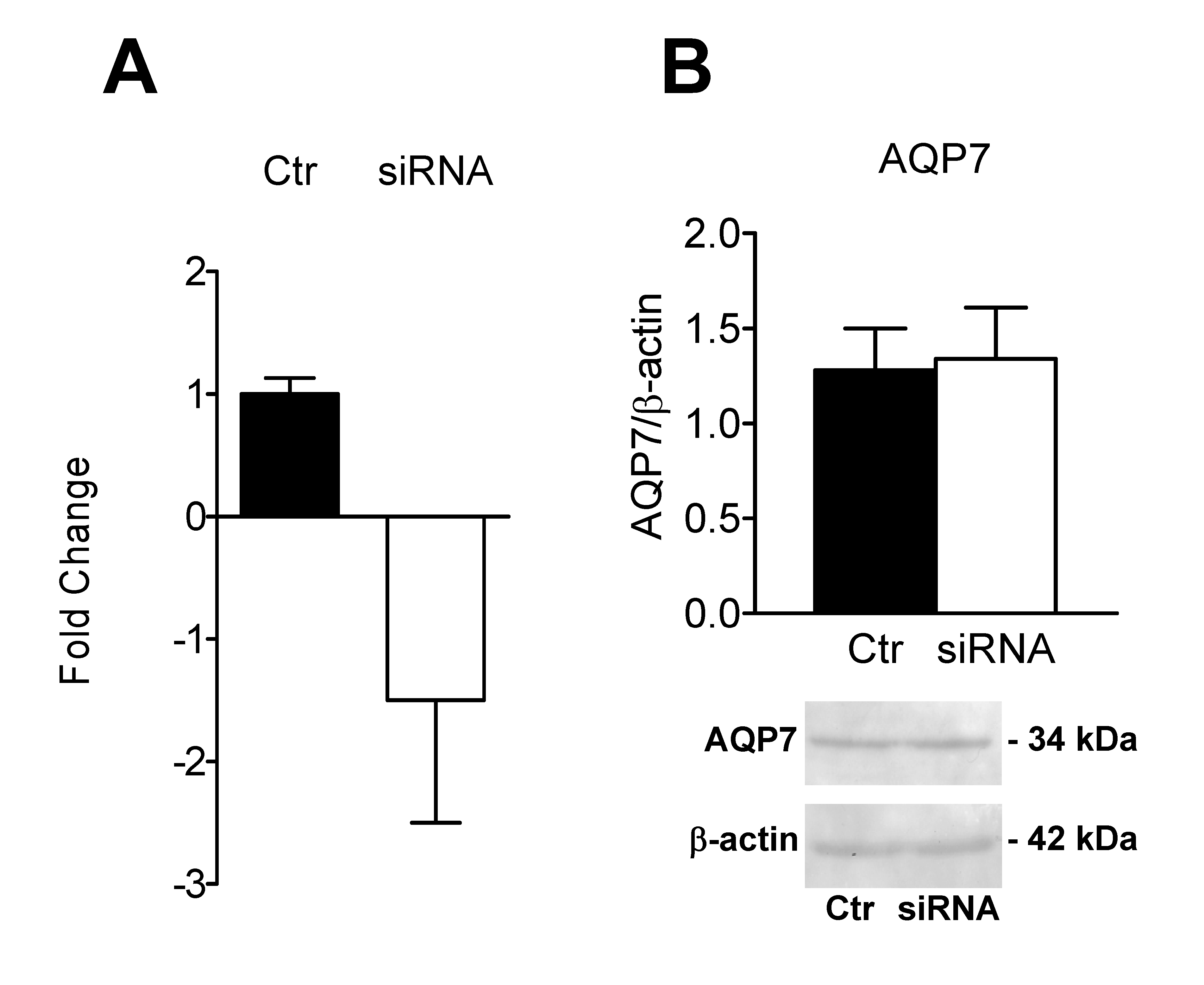

Supplement: Figure S1 — Aquaporin-7 (AQP7) expression in aquaporin-10 (AQP10) silenced human differentiated adipocytes. AQP10 short interfering RNA (siRNA) and scrambled siRNA (Ctr) were transfected in differentiated adipocytes as described in Materials and methods. A, AQP7 mRNA levels were measured by real-time RT-PCR relative to the β-actin internal standard and the values obtained were reported as fold change (see Materials and Methods section). Bars represent the mean ± SEM of at least 4 different experiments each from different RNA extracts. AQP7 transcript was unmodified in silenced differentiated adipocytes (siRNA) compared to controls (scrambled; Ctr) (P = 0.186; Student’s t test). B, Western blot and densitometry demonstrate that also AQP7 protein was unmodified (P = 0.872; Student’s t test). Blots representative of three were shown (B, lower pannel). The same blots were stripped and re-probed with anti-β-actin antibody. Bands of the expected molecular weights were shown and acquired with the Image Master VDS (GE Healthcare Life Sciences, Italy). Densitometric analysis of the bands was performed by Total Lab V 1.11 computer program (GE Healthcare Life Sciences, Italy) and the results were normalized to the corresponding β-actin (B, upper panel). (TIF) [file pone.0054474.s001.tif]
